# Inclusion of grape marc in dairy cattle rations alters the bovine milk proteome

**DOI:** 10.1017/S0022029919000372

**Published:** 2019-05

**Authors:** Richard A. Scuderi, David B. Ebenstein, Ying-Wai Lam, Jana Kraft, Sabrina L. Greenwood

**Affiliations:** 1 Department of Animal and Veterinary Sciences, The University of Vermont, 570 Main St, Burlington, VT 05405, USA; 2 Vermont Genetics Network Proteomics Facility, The University of Vermont, 109 Carrigan Dr, Burlington, VT 05405, USA; 3 Department of Biology, The University of Vermont, 109 Carrigan Dr, Burlington, VT 05405, USA

**Keywords:** By-product, condensed tannins, nitrogen metabolism, ruminant nutrition

## Abstract

Grape marc (GPM) is a viticulture by-product that is rich in secondary compounds, including condensed tannins (CT), and is used as a supplement in livestock feeding practices. The aim of this study was to determine whether feeding GPM to lactating dairy cows would alter the milk proteome through changes in nitrogen (N) partitioning. Ten lactating Holstein cows were fed a total mixed ration (TMR) top-dressed with either 1.5 kg dry matter (DM)/cow/day GPM (GPM group; *n* = 5) or 2.0 kg DM/cow/day of a 50:50 beet pulp: soy hulls mix (control group; *n* = 5). Characterization of N partitioning and calculation of N partitioning was completed through analysis of plasma urea-N, urine, feces, and milk urea-N. Milk samples were collected for general composition analysis, HPLC quantification of the high abundance milk proteins (including casein isoforms, α-lactalbumin, and β-lactoglobulin) and liquid chromatography tandem mass spectrometry (LC-MS/MS) analysis of the low abundance protein enriched milk fraction. No differences in DMI, N parameters, or calculated N partitioning were observed across treatments. Dietary treatment did not affect milk yield, milk protein or fat content or yield, or the concentrations of high abundance milk proteins quantified by HPLC analysis. Of the 127 milk proteins that were identified by LC-MS/MS analysis, 16 were affected by treatment, including plasma proteins and proteins associated with the blood-milk barrier, suggesting changes in mammary passage. Immunomodulatory proteins, including butyrophilin subfamily 1 member 1A and serum amyloid A protein, were higher in milk from GPM-fed cows. Heightened abundance of bioactive proteins in milk caused by dietary-induced shifts in mammary passage could be a feasible method to enhance the healthfulness of milk for both the milk-fed calf and human consumer. Additionally, the proteome shifts observed in this trial could provide a starting point for the identification of biomarkers suitable for use as indicators of mammary function.

In bovine milk, there are a number of bioactive proteins and peptides, including those with growth, cellular signaling, immunomodulation, antimicrobial, and anti-cancer functions (Korhonen, 2009; Mills *et al*., 2011). Stage of lactation, genetics and nutrition are known to influence the composition of the bovine milk proteome (Le *et al*., 2011; Yang *et al*., 2015; Tacoma *et al*., 2016). Characterizing the milk proteome could yield three important contributions to the global dairy industry: identification and validation of milk protein biomarkers to identify physiologic perturbances or imbalances, development of milk products rich in healthful bioactive milk proteins for the consumer market and enhanced quality of colostrum for calf development (Greenwood and Honan, 2019). Nutritional factors that affect the milk proteome are of particular interest since the proteome appears to be reactive to diet profile and animal nutrition is a variable and important component in farm management. Recently, Li *et al*. (2015) observed that offering dairy cattle varying degrees of ruminally-available diets by way of energy and rumen degradable protein (RDP) resulted in an increase in specific milk proteins, including zinc-α−2-glycoprotein (ZAG) and alpha-lactalbumin (α-LA), implying that diet impacts the bovine milk proteome. Earlier, Christian *et al*. (1999) observed changes in milk casein fractions when cows were fed diets with varying degrees of lupin and wheat, effectively altering the proportions of ruminally-available protein and energy. The work done by Christian *et al*. (1999) and Li *et al*. (2015) highlights the potential for altering the proteomic composition of bovine milk as a result of rumen available protein, likely by altering post-absorptive nitrogen (N) metabolism.

Grape marc (GPM) is a byproduct of the viticulture industry consisting of the remaining skins, stems, and seeds of the grapes after pressing for wine. The wine-making industry has sought out ways to utilize the byproduct, however, much of the grape pomace is composted and considered to be a pollutant (Tsiplakou and Zervas, 2008; Santos *et al*., 2014). As a result, GPM has been incorporated into livestock feeding practices, including the dairy industry, due to its low-cost and regional availability (Nudda *et al*., 2015; Manso *et al*., 2016). Supplementing GPM in dairy diets also appears to have no impact on milk production. Nudda *et al*. (2015) reported no change in milk production when feeding 300 g GPM, and Santos *et al*. (2014) also reported no differences in milk composition when diets included ensiled GPM at amounts of 50, 75, and 100 g/kg DM (Santos *et al*., 2014; Nudda *et al*., 2015).

GPM contains polyphenolic compounds, including condensed tannin(s) (CT) that provide an array of bioactivities (Waghorn, 2008; Nudda *et al*., 2015). By binding to proteins in the rumen, CT-inclusive diets alter the N metabolism in ruminants by decreasing the RDP fraction, and thereby increasing the RUP fraction available for proteolysis by the animal in the abomasum and small intestine (Waghorn, 2008). Shifting sites of N uptake, as well as the amount and profile of protein available in the small intestine for absorption also impacts animal-derived products, such as milk, as has been observed when feeding other CT-rich feeds, including birdsfoot trefoil (Woodward *et al*., 2009), red clover (Lee *et al*., 2009), and chicory (Totty *et al*., 2013) for example (Acamovic and Brooker, 2005).

While research has highlighted the potential benefits of GPM inclusion on increasing bioactive fatty acids in the milk (Manso *et al*., 2016) and modulating hepatic inflammation (Gessner *et al*., 2015) in the dairy cow, none have investigated the impact of GPM feeding on the milk proteome. Given the wide-spread availability of GPM, and GPM's known ability to impact post-absorptive metabolism in the dairy cow, this original research could yield insight on a feasible mechanism to alter the milk proteome and provide an important application to the dairy industry. The hypothesis of the research described herein was that dietary supplementation of GPM to lactating dairy cows would alter the bovine milk proteome, including ZAG and α-LA, *via* changes in N partitioning. The objectives of this study were to (i) determine the milk protein profile using proteomic approaches and summarize ontological functions of the identified proteome, and (ii) assess concurrent changes in N partitioning by measuring indicators of N status in milk, plasma, urine, and feces.

## Materials and methods

The experiments reported here were done in accordance with the Institutional Animal Care and Use Act (IACUC) at The University of Vermont (Burlington, VT). A power analysis performed using previous literature by Li *et al*. (2015), which reported the impacts of dietary treatments on milk ZAG abundance, confirmed the appropriate animal number per treatment that was used in this study (comparing treatments B and D at 80% power with 30% coefficient of variation and 70% treatment difference).

### Animals, diet, and experimental design

Ten mid-lactation Holstein cows were paired by parity (1.2 ± 0.4), DIM (147 ± 37 d), and daily milk yield (41.3 ± 5.4 kg) and stratified within pair across two treatments for a 28 d trial. Cows were not genotyped prior to this experiment, so this was not used as a parameter to assign treatments. Cows within each pair were assigned to either control (*n* = 5) or treatment (*n* = 5) diets. The experiment consisted of a 21 d adaptation period in order to allow cows to adapt to their new diet, followed by a 7 d sample collection period. All cows were housed in tie-stalls at the Paul R. Miller Research and Education Center (The University of Vermont, Burlington, VT). Cows had free access to water and were fed a total mixed ration TMR diet. All cows were milked at 4:00 h and 16:00 h daily. Each cow was given their entire weighed daily base ration, which included grass silage (17.6% of DM), corn silage (37.7% of DM), and a mash (7.9% of DM), once daily at 5:00 h. The remaining portion of the base ration (36.8% of DM) was a concentrate pellet, which was mixed into the base ration four times daily (3:30, 10:00, 15:30, and 22:00 h). Cows assigned to the control diet received a ration containing a beet pulp: soy hulls (50:50, BP) mixture at 2.0 kg DM/cow/d, while cows assigned to the treatment diet received a ration containing GPM at 1.5 kg DM/cow/d. Rations were fed at 5:00 h for the duration of the 28 d trial. GPM was sourced from a local vineyard (Shelburne Vineyard, Shelburne, VT), and stored on-farm under a covered landing in 1 ton harvest bags.

Diet refusals from each cow were collected daily before feeding (5:00 h) and weighed, then a subsample was stored at −20 °C for further analysis. Samples were later dried at 65 °C for 48–72 h for determination of individual DMI. Feed samples were also collected weekly across the 28 d period and composited within feedstuff for wet-chemistry analysis (Dairy One Lab, Ithaca, NY; Supplementary Table S1).

GPM samples, collected once weekly, were stored at −20 °C until later analysis of CT content as per methods previously described (Sarneckis *et al*., 2006; Mercurio *et al*., 2007) with minor modifications. Briefly, a composite of the weekly samples was created and then blended using a Bella Rocket Blender (Sensio, Montreal, QC, Canada), and homogenized using a Qiagen TissueLyserII at 30 Hz for 3 min (Qiagen, Hilden, Germany). Ten milliliters of 50% ethanol solution were then added to 1 g of the homogenate, vortexed, and placed on a shaker for 1 h. Centrifugation immediately followed at 4695 × ***g*** for 10 min at room temperature. The supernatant was removed and analyzed using a Methyl Cellulose Precipitable Tannin Assay (MCP) as previously outlined (Sarneckis *et al*., 2006; Mercurio *et al*., 2007).

### Plasma

Blood samples were collected from the coccygeal vessel in heparinized and ethylenediaminetetraacetic acid-coated (EDTA) vacutainers (BD, Franklin Lakes, NJ) after AM and PM milking during the covariate period (d 0), and d 21 and 28 of the sample collection period. Samples were immediately placed on ice and centrifuged at 3000 × ***g*** for 15 min at 4 °C. Plasma was harvested and stored at −20 °C until further analysis. Samples were analyzed using commercially available kits for plasma urea nitrogen (PUN; Teco Diagnostics, Anaheim, CA) concentrations.

### Urine and feces

Total urine and fecal collections from each cow were completed on d 28. Urine was collected using modified urine cup collectors as previously described (Lascano *et al*., 2010). Briefly, cows were fitted with urine collection devices attached to 40 l carboys containing 100% sulfuric acid (H_2_SO_4_; Fisher Scientific, Pittsburgh, PA) to acidify the urine to a pH <4 as it was collected from each animal. The H_2_SO_4_ was incrementally added to the carboys during the sampling period, totaling 350 ml. At the end of the 24 h, the urine collected from each animal was mixed thoroughly, the total weight was recorded, and a subsample was collected. Feces was collected *via* free-catch onto tarps behind the cows, and transferred to holding bins for each animal during the 24 h collection period. The fecal matter was mixed thoroughly, the total weight was recorded, and a subsample for each cow was collected. All urine and fecal subsamples were stored at −20 °C until being submitted for wet-chemistry analysis (Dairy One, Ithaca, NY). Endpoint measures included urine urea, urine ammonia, urine CP, fecal N, and fecal ammonia N.

### Milk sampling

Milk yield was recorded at each milking, and samples were collected from each cow using continuous in-line samplers at AM and PM milkings. Milk samples were collected at AM and PM milking from each cow on d 0 and again at AM and PM milking on three days during the experimental period (d 25, 27, and 28) for further analyses. One set of milk samples were transferred into tubes containing the preservative bronopol at the time of milking, stored at 4 °C, and submitted for commercial analysis of milk fat, protein, somatic cell count (SCC) and milk urea nitrogen (MUN) content to the DHIA (Lancaster, PA). The two additional samples collected, one for HPLC analysis (5 ml) and one liquid chromatography tandem mass spectrometry (LC-MS/MS) analysis (30 ml), were immediately placed into a dry ice/ethanol bath on-farm before being stored at −20 °C (HPLC analysis) and −80 °C (LC-MS/MS analysis).

### Analysis of high-abundance milk proteins using HPLC methodology

Milk samples collected for HPLC analysis of the high-abundance milk proteins, including α-s1, α-s2, β and κ-caseins (CAS), α-LA, and the A and B variants of β-lactoglobulin (β-LGA, and β-LGB, respectively) were thawed overnight at 4 °C. Samples collected during d 0 were pooled within cow according to milk yield (totaling 10 composite samples, one per cow), while the samples collected at AM and PM milking during the experimental period (d 25, 27, and 28) were pooled within cow as a proportion of milk yield (totaling 10 composite samples, one per cow). Samples were then centrifuged at 4000 × ***g*** for 10 min at 4 °C to allow for separation of the cream layer. The skim milk fraction was processed and analyzed using HPLC as previously described (Bordin *et al*., 2001; Tacoma *et al*., 2016). This methodology does not include quantification of γ-casein and this cleavage fragment was not determined in this study. Briefly, a reducing buffer containing dithiothreitol (DTT), 6 M guanidine-HCl, and 5 mM trisodium citrate in water was added to each sample. Samples were then vortexed and left in the fridge to incubate overnight. After incubation, a volume of buffer without the reducing agent DTT was added to each sample and the solution was filtered through a 0.45 µm syringe filter (Sartorious. Göttingen, Germany) into a borosilicate test tube. The filtrate was transferred to autosampler vials for subsequent HPLC analysis (Shimadzu Corporation, Kyoto Japan). Separations were completed according to methods previously described by Bordin *et al*. (2001) on a C_4_ reversed-phase microbore analytical column (150 × 2.1 mm, 300 Å pore diameter and 5 µm particle size, Yydac 214 MS, Grace Davison, MD, USA).

### Preparation of low-abundance protein enriched milk fraction

Milk samples collected at AM and PM milking during the experimental period (d 25, 27, and 28) for the identification of low-abundance enriched proteins through LC-MS/MS analysis were thawed overnight at 4 °C and were composited within cow by milk yield as described above for HPLC analysis, yielding 10 composite samples, one per cow. Protein fractionation and enrichment were performed as previously described (Tacoma *et al*., 2016). Briefly, a protease inhibitor (Protease Inhibitor Cocktail, SiGPMa, Milwaukee, WI) was added at 0.24 ml per g of protein, followed by centrifugation at 4000 × ***g*** for 10 min at 4 °C to allow for separation of the cream layer. Skim milk samples were depleted of casein by calcium dichloride precipitation followed by ultracentrifugation at 189 000 × ***g*** for 70 min at 4 °C. The supernatant was stored at −80 °C prior to lyophilization and reconstitution in PBS. The protein concentration of the reconstituted samples was determined using the bicinchoninic acid assay (BCA; Pierce, Rockford, IL) using bovine serum albumin as the standard. Samples were enriched using a ProteoMiner kit (BioRad, Hercules, CA) as per manufacturer's instructions. Eluted samples were analyzed for protein concentration using BCA. 1 µg of *Saccharomyces cerevisiae* GAPDH (Glyceraldehyde-3-phosphate Dehydrogenase; SiGPMa-Aldrich, St. Louis, MO) was added to 99 µg of each sample, and each of the samples (100 µg total) were digested with trypsin followed by labeling using isobaric Tandem Mass Tags (TMT) as per manufacturer's instructions (product #90113; Thermo Scientific, Rockford, IL). Samples were then combined in equal parts, and the 10plex was kept at −80 °C until subsequent LC-MS/MS.

### Liquid chromatography—mass spectrometry

Four microliters of the TMT reaction mixture were dried under vacuum and labeled peptides were resuspended in 10 µl of 2.5% acetonitrile (CH_3_CN) and 2.5% formic acid (FA) in water for subsequent liquid chromatography-mass spectrometry (LC-MS) analysis similar to that described by Tacoma *et al*. (2017). Briefly, LC-MS-based peptide identification and quantification was performed on the Q-Exactive mass spectrometer coupled to an EASY-nLC (Thermo Fisher Scientific, Waltham, MA). Five microliters of the sample was loaded onto a 100 µm × 120 mm capillary column packed with Halo C18 (2.7 µm particle size, 90 nm pore size, Michrom Bioresources, CA, USA) at a flow rate of 300 nl/min. Peptides were separated using a gradient of 2.5–35% CH_3_CN/0.1% FA over 150 min, 35–100% CH_3_CN/0.1% FA in 1 min and then 100% CH_3_CN/0.1% FA for 8 min, followed by an immediate return to 2.5% CH_3_CN/0.1% FA and a hold at 2.5% CH_3_CN/0.1% FA. A nanospray ionization source introduced the peptides into the mass spectrometer through the use of a laser pulled ~3 µm orifice with a spray voltage of 2.0 kV. Mass spectrometry data was acquired in a data-dependent manner using ‘Top 10’ acquisition mode with lock mass function activated (*m/z* 371.1012; use lock masses: best; lock mass injection: full MS), in which a survey scan from *m/z* 350–1600 at 70 000 resolution (AGC target 1e^6^; max IT 100 ms; profile mode). Following data acquisition, 10 higher-energy collisional dissociation MS/MS scans were performed on the most abundant ions at 35 000 resolution (AGC target 1e^5^; max IT 100 ms; profile mode). An isolation width of 1.2 *m/z* and a normalized collisional energy of 35% was used to obtain MS/MS scans, and dynamic exclusion was enabled (peptide match: preferred; exclude isotopes: on; underfill ratio: 1%; exclusion duration: 30 s). SEQUEST and Mascot search engines were used for the subsequent product ion spectra on Proteome Discoverer 1.4 (Thermo Fisher Scientific, Waltham, MA, USA) against a curated Bovine Uniprot (*Bos taurus* database; UP000009136; 24 346 entries; downloaded Dec. 9, 2015) with sequences in forward and reverse orientations. To verify effective tryptic digestion and subsequent labeling of peptides, the product ion spectra were re-searched against a *Saccharomyces cerevisiae* database. Search parameters were as follows: full trypsin enzymatic activity, maximum missed cleavages = 2, and peptides MW between 350 to 5000; mass tolerance at 20 ppm for precursor ions and 0.02 Da for fragment ions, dynamic modifications on methionines (+15.9949 Da: oxidation), Dynamic TMT6plex modification (The TMT6plex and TMT10plex have the same isobaric mass) on N-termini and lysines (229.163 Da), as well as static modification on cysteines (+57.021 Da). Percolator node was used to limit the false positive (FP) rates to less than 1% in the data set. Reporter Ion Quantification Node in Proteome Discoverer 1.4 was used for quantification purposes. All of the acquired protein identification and quantification information (<1% FP; with protein grouping enabled) was exported to Excel spreadsheets. Relative fold-change values of the proteins identified within each animal were compared against values from each of the control cows for data generated using both the *Bos taurus* and *Saccharomyces cerevisiae* databases. *Saccharomyces cerevisiae* GAPDH (accession numbers: P00359 and P00360) and *Bos taurus* GAPDH (accession number: P10096) search results shared one common amino acid sequence (LTGPMAFR); therefore, protein P10096 was excluded from bioinformatics.

### Bioinformatics

Milk proteins identified by LC-MS/MS analysis that were classified as uncharacterized through Proteome Discoverer 1.4 were identified using basic local alignment search tool (BLAST) (Camacho *et al*., 2009). Proteins that were identified as affected by dietary treatment through statistical analysis were matched to their associated annotated functions using gene ontology (GO) through The PANTHER Classification System (Mi *et al*., 2017). Proteins were annotated to their biological process, molecular function, cellular component, and protein class and graphed in Prism 7 (GraphPad Software Inc., La Jolla, CA) according to their percent of gene hits against total number of function hits as calculated from PANTHER.

### Calculation of nitrogen intake, excretion, and retention

Daily N intake (g) of each cow was calculated by multiplying DMI (g) by the CP content (% of DM) of the feed, and dividing by 6.25 to determine g N/d intake. Daily fecal N output (g) of each cow was calculated by multiplying the weight of feces collected after 24 h by the fecal *N* %. Daily urine *N* output (g) of each cow was obtained by multiplying the weight of urine collected after 24 h by the urine CP %, and dividing by 6.25 to obtain the estimated g N/d excretion in urine. Daily milk *N* output (g) of each cow was calculated by dividing the milk protein yield (g) of the cow by 6.38 to obtain g N/d secretion in milk. The *N* retention of each cow was then calculated by subtracting the g N/d excreted in urine, feces and milk from the g N/d intake.

### Statistical analysis

The PROC MIXED procedure was used in SAS version 9.4 (SAS Institute, Cary, NC) to perform repeated measures ANOVA on DMI, milk components, and plasma results. Treatment, day, and a day × treatment interaction were used as fixed effects, and d 0 was included as a covariate in each of these models. PROC MIXED model was also used in SAS version 9.4 for analysis of the endpoint values for high-abundance proteins and urine and fecal parameters with treatment included as a fixed effect. Fold change of each milk protein identified by LC-MS/MS was calculated relative to each control sample, and milk proteins were then statistically analyzed as repeated measures with treatment included as a fixed effect. Conditional formatting was performed in Excel (v.14.2.2.) to generate a three-way color scale heat map hybridized with the table listing relative fold change values. Significant differences were declared if *P* ≤ 0.05.

## Results

### Diet, milk yield and components

There were no differences in DMI between treatment groups (Supplementary Table S2). Total milk yield (kg/d), milk components (% or kg/d), and SCC were not different across treatments (Supplementary Table S2).

### Nitrogen parameters and N partitioning

There was no difference in N intake (g N/d), PUN concentrations, MUN concentrations, urine N parameters, fecal N parameters, or calculated g N/d in urine, feces, or milk, or g N/d retained across treatments (Supplementary Table S3).

### Milk proteome and bioinformatics analysis

**Fig. 1.**
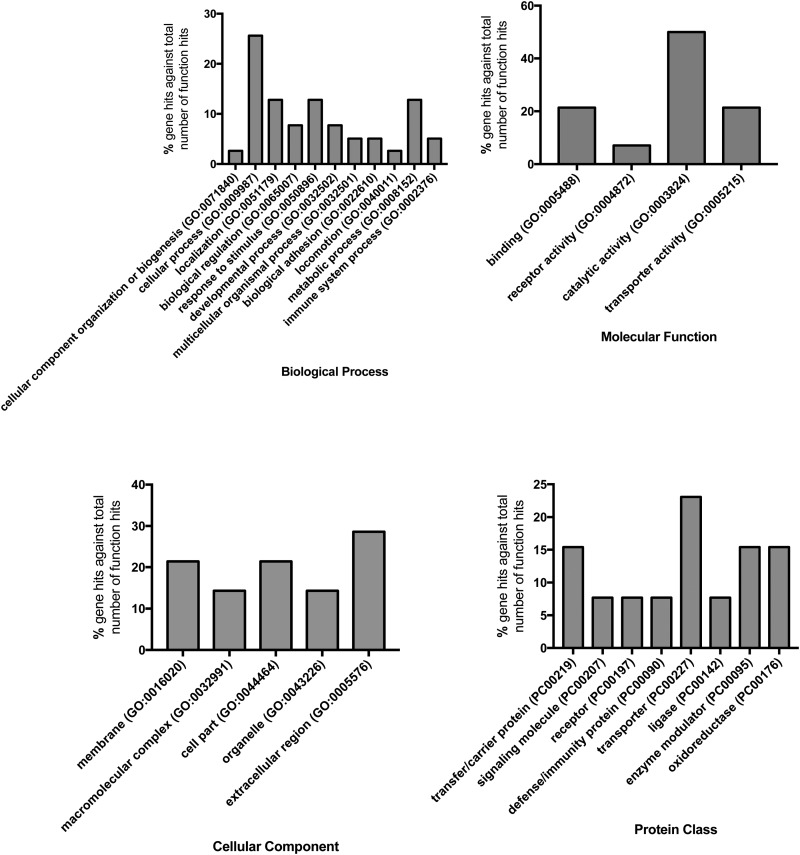
Gene ontology (GO) representing the biological processes, molecular functions, cellular components, and protein classes of proteins identified by LC-MS/MS that were different between treatment groups.

There were no differences in the milk concentrations of high abundance proteins α-s1, α-s2, β or κ- CAS, α-LA, β-LGA, or β-LGB across treatment groups (Table 1). A total of 127 proteins were identified using LC-MS/MS techniques (Supplementary Table S4), and of those, 16 were affected by treatment (Table 2). Gene ontology analysis of the 16 affected proteins revealed cellular process (GO term: 0009987; 25.6%) as the most prominent term for biological process, followed by metabolic process (GO term: 0008152; 12.8%), response to stimulus (GO term: 0050896; 12.8%), and localization (GO term: 0051179; 12.8%; Fig. 1, Supplementary Table S5). Accounting for 50% of the 16 proteins, catalytic activity (GO term: 0003824) was the most annotated molecular function term. Cellular component analysis categorized 28.6% of the proteins as extracellular (GO term: 0005576) and an additional 21.4% were identified to be of membrane origin (GO term: 0016020). Additionally, the most prominent protein classes included: transporter (GO term: PC00227; 23.1%), oxidoreductase (GO term: PC00176; 15.4%), transfer/ carrier protein (GO term: PC00219; 15.4%), and enzyme modulator (GO term: PC00095; 15.4%) classifications.

**Table 1.** High-abundance protein concentrations from lactating Holstein dairy cows fed a diet supplemented with either grape marc (GPM) or beet pulp: soy hulls mixture (control)

Milk protein (mg/ml skim milk)	Control	GPM	se	*P*-value
α-S1 CAS	13.02	13.42	0.22	0.24
α-S2 CAS	1.78	1.78	0.05	1.00
β-CAS	17.94	18.48	0.28	0.23
κ-CAS	5.55	5.65	0.09	0.43
α-LA	3.01	3.00	0.01	0.55
β-LGA	3.20	3.16	0.07	0.70
β-LGB	3.39	3.33	0.02	0.10

se, standard error; CAS, casein; β-LGA, β-Lactoglobulin variant A; β-LGB, β-Lactoglobulin variant B.

Least square means reported for Control and GPM groups.

**Table 2.** Low-abundance proteins identified in milk samples at significantly different relative-abundances collected from lactating Holstein dairy cows fed a diet supplemented with either grape marc (GPM) or beet pulp: soy hulls mixture (control), depicted as a hybridized heatmap

Accession number	Protein	Control	GPM	se	*P-*Value
Q03247	Apolipoprotein E	1.01	1.43	0.083	0.002
Q3MHN2	Complement component C9	1.06	0.73	0.086	0.012
P17697	Clusterin	1.04	1.42	0.073	0.014
P18892	Butyrophilin subfamily 1 member A1	1.17	2.13	0.319	0.015
F1MM32	Sulfhydryl oxidase	1.07	0.77	0.088	0.019
Q4GZT4	ATP-binding cassette sub-family G member 2	1.11	1.62	0.210	0.021
F1MIT3	von Willebrand factor A domain-containing protein 8	1.41	13.97	3.519	0.022
M0QW03	TPA: prolactin-like protein	1.06	1.53	0.159	0.025
P01035	Cystatin-C	1.17	0.67	0.126	0.030
P02769	Serum albumin	1.30	0.66	0.180	0.032
F1MMW8	Serum amyloid A protein	1.09	1.76	0.235	0.033
F1MNV5	Kininogen-1	1.15	0.75	0.131	0.036
P26201	Platelet glycoprotein 4	1.44	2.79	0.518	0.040
F1N6D4	Sodium-dependent phosphate transport protein 2B	1.47	2.90	0.565	0.042
F1MUP9	Synaptic vesicle membrane protein VAT-1 homolog	1.02	1.26	0.081	0.044
Q0V8M0	Protein KRI1 homolog	1.02	1.16	0.033	0.046

se, standard error.

Least square means reported for Control and GPM groups expressed as relative-abundance, with intensity of blue increasing as relative abundance increases above 1.0 and intensity of red increasing as relative abundance decreases below 1.0.

## Discussion

In this experiment, feeding GPM to cows did not result in any changes in their N status; however, 16 milk proteins were affected by dietary treatment. Of the 16 milk proteins altered by dietary treatment, 28.6% were of extracellular origin, with another 21% of membrane origin (Fig. 1). In terms of protein classification, 15% of the treatment-affected proteins were annotated as transfer/carrier proteins, which include proteins that carry substances and do not involve transmembrane transport, and 23% of the proteins were annotated as transporter proteins, which do include proteins with transmembrane activity (Fig. 1; Mi *et al.* 2017). While the gene ontology profile of the greater proteome identified in this experiment was in line with the average skim milk proteome profile outlined in a recent review by Greenwood and Honan (2019), the gene ontology of proteins affected by diet in this experiment appears to contain more proteins with the cellular component classification of membrane origin and the protein class of transporter, suggesting these specific groups of proteins were disproportionately affected by feeding GPM.

Proteins known to be present in blood, including von Willebrand factor A domain-containing protein, platelet glycoprotein 4, kininogen-1 and serum albumin, were all identified in milk samples in the current experiment, and their abundance was affected by diet. While all of these proteins have previously been identified in ProteoMiner-treated milk fractions (Molinari *et al*., 2012; Tacoma *et al*., 2016), the current finding that GPM treatment affected their relative abundance in milk suggests that perhaps paracellular or transcytotic passage of these extracellular proteins across the mammary epithelium was changed, which are known mechanisms of blood-milk protein connectivity (Shennan and Peaker, 2000; Monks and Neville, 2004; Kobayashi *et al*., 2013). Interestingly, butyrophilin subfamily 1 member A1 (BTN), apolipoprotein E, clusterin, platelet glycoprotein 4, and sodium-dependent phosphate transport protein 2B were all higher in relative abundance in milk from GPM-fed cows as compared to control cows. These same proteins have also been identified as potential proteins of interest in mastitis research, which is another circumstance of shifted passage dynamics between the blood and milk. Zhang *et al*. (2015) identified a relationship between SCC score of the milk and BTN, apolipoprotein E, clusterin, kininogen-1, sodium dependent phosphate transport protein 2B, and platelet glycoprotein 4 abundance. In the experiment by Zhang *et al*. (2015), none of these proteins followed a positive linear correlation with increasing SCC content of milk, but instead these proteins all peaked in abundance in the samples containing between 4 × 10^5^ and 7.5 × 10^5^ cells/l and were often present at significantly lower abundances in the milk with the highest SCC content (10^6^ cells/ml) compared to the low SCC milk sample. Our observation of lower serum albumin in milk from GPM-fed cows is contradictory to the well reported increase of serum albumin in milk, including by Zhang *et al*. (2015), during mastitis conditions; however, this could be a result of our enrichment method, specifically the inclusion of ProteoMiner treatment in our workflow, and hence interpretation of this specific protein should be done with caution. While Zhang *et al*. (2015) also identified ATP-binding cassette sub-family G member 2 protein in milk, they did not observe any impact of SCC on this protein; however, our trial identified a higher abundance of this protein in milk as a result of GPM feeding. Previous research focused on this protein, also known as efflux transporter ABCG2, has highlighted its importance in blood-tissue barrier function at the apical membrane of numerous tissues (Vlaming *et al*., 2009), with a more recent report confirming its importance in maintaining the blood-milk barrier at the apical membrane of alveolar epithelial cells in cattle and the sensitivity of this protein to drug compounds (Mahnke *et al*., 2016). A higher abundance of this protein in this current study could be another indicator that supports the concept of a change in blood-milk passage.

In terms of shifts in bioactive proteins, BTN is also an immunomodulatory bioactive protein (Spitsberg, 2005) and was present at higher relative abundance in milk from GPM-fed cows relative to milk from controls (2.13 fold increase in BTN). Though BTN is the major protein associated with the milk-fat globule membrane (MFGM) (Spitsberg, 2005), its presence in non-fat milk fractions has previously been reported (Nissen *et al*., 2013), and is in agreement with the current study. Characterization of BTN has led to a better understanding of the numerous functions of BTN family members in addition to BTN's contribution to milk fat globule stability, including an important role in immune cell activation (Arnett and Viney, 2014). This increase in abundance of BTN occurred alongside a higher relative abundance (1.76 fold increase) of a second immunomodulatory bioactive protein, serum amyloid A protein (SAA3) (Mills *et al*., 2011). SAA3 in blood has been classically known as a marker of inflammation; however, there is also evidence suggesting direct antimicrobial activity (Molenaar *et al*., 2009; Mills *et al*., 2011). It is now accepted that SAA3 also originates from the mammary gland, and it has been shown that mammary-derived SAA3 exhibits an extra-mammary protective response against microbial infection (Molenaar *et al*., 2009). It has been further proposed that the antimicrobial role of SAA3 is included in a more generalized host response that affects the binding properties of pathogens (Mills *et al*., 2011).

Kininogen-1 and cystatin-C have both previously been identified in milk and their milk-derived purified forms have both been used to stimulate osteoblastic cell proliferation in vitro using MC3T3-E1 cells (Yamamura *et al*., 2000; Yasueda *et al*., 2018). Both of these proteins were present at lower abundance in milk from GPM-fed cows compared to control cows. From an industry perspective, it would be interesting to investigate whether these milk proteins could be used for biomarkers of osteoblast function in the lactating cow or whether dietary-induced shifts in the abundance of these proteins in milk could impact osteoblast-related bioactivity of the milk once ingested by the milk-fed calf or human consumer.

Other bioactive proteins whose abundance in milk was lowered by feeding GPM included complement component C9 and sulfhydryl oxidase. While these proteins have already been identified in bovine milk and their bioactivity has been proposed (Korhonen *et al*., 2000; Jaje *et al*., 2007), their roles and regulation are less clear. Complement component C9 appears to have a clear role in innate immunity (Korhonen *et al*., 2000), but also appears to be involved in bactericidal and hemolytic activity in milk (Rainard, 2003). Sulfhydryl oxidases are plentiful, and some potentially have a role in innate immunity geared toward offspring protection (Isaacs *et al*., 1984), but ultimately this enzyme oxidizes free sulfhydryl groups by using oxygen as an electron acceptor with hydrogen peroxide being produced as a result. Hydrogen peroxide production in milk is reduced in other periods of higher blood and milk interactivity, including mammary inflammation caused by mastitis (Sakai *et al*., 2008), and hence a decrease in sulfhydryl oxidase abundance is in line with our suggestion that the blood-milk barrier may be affected by feeding GPM.

While shifts in N partitioning do not appear to be the causal factor, other diet parameters such as carbohydrate fraction or secondary compounds could be influencing the milk proteome. Cows fed GPM also consumed approximately 400 g/d more lignin from their supplement as compared to the control cows (Supplementary Table S1). It is plausible that lignin could have had a small impact on DMI and nutrient uptake, ultimately impacting mammary nutrient availability. A second possible mechanism of diet-induced milk proteome shifts in the current study is through other phytochemicals apart from CT, such as anthocyanins or phytoestrogens, which are rich in grape pomace (Yi *et al*., 2009) but were not measured in this study. Anthocyanins have been documented to have several human health impacts, including immunomodulation (He and Giusti, 2010), which could in part explain the observed differences in the milk proteome of GPM-fed cows compared to control cows. Based on research by Yi *et al*. (2009), the anthocyanin content of the GPM would be approximately 0.1%, equating to an intake of roughly 1.8 g anthocyanins per treatment cow per day in the current study. While not all anthocyanins are phytoestrogenic (Welch *et al.* 2008), grape products do contain estrogenic compounds (Kopp, 1998). Phytoestrogens are known to weaken the blood-milk barrier in mammary epithelial cells in vitro through both a decrease in cell viability and transepithelial resistance, ultimately leading to increased epithelial barrier permeability (Tsugami *et al*., 2017), which would coincide with our observation that a large portion of the milk proteins affected by dietary GPM inclusion have known functions and roles in maintaining the blood-milk barrier.

In conclusion, sixteen proteins in the low-abundance enriched milk protein fraction were affected by diet despite the low CT content of the GPM and lack of evidence of any shifts in N partitioning as result of GPM intake. While not measured in this experiment, this proteome shift could possibly be a result of anthocyanins or phytoestrogens in GPM. Gene ontology of these affected milk proteins suggested that many of these proteins are not of intracellular origin, and instead many of these proteins are known to be associated with blood or the blood-milk barrier. This study does support the suggestion that the milk proteome can be affected by diet, particularly through manipulation of secondary compounds within the diet, and that the dairy industry could focus on diet as a mechanism to alter the milk proteome. A feasible use for this research is for identification and validation of specific proteins in milk that can be used as biomarkers to identify non-mastitic shifts in mammary function. Furthermore, our observation of shifts in known bioactive proteins is also important from the perspective of the consumer market, with dietary manipulation being a potential means to enhance the nutritional value of milk for both the milk-fed calf and the human consumer.

## Supplementary material

For supplementary material accompanying this paper visit http://dx.doi.org/10.1017/S0022029919000372. click here to view supplementary material
