# Supplementary material for: Inclusion of grape marc in dairy cattle rations alters the bovine milk proteome
Source: J Dairy Res. 2019 May;86(2):154–61. doi: 10.1017/S0022029919000372 (PMC7033658; doi:10.1017/S0022029919000372)
Supplement: Supplementary file 1 [file S0022029919000372sup001.pdf]

Inclusion of grape marc in dairy cattle rations alters the bovine milk proteome

Richard A. Scuderi, David B. Ebenstein, Ying-Wai Lam, Jana Kraft and Sabrina L. Greenwood

SUPPLEMENTARY FILE

Supplementary Table S1. Chemical composition of supplements and diet

	Control	GPM
Supplement chemical composition		
Dry Matter (%)	90,1	30,2
Crude Protein (% DM)	9,9	12,8
ADF (% DM)	41,3	44,2
aNDFom (% DM)	50,5	53,2
Lignin (% DM)	3	30,2
NFC (% DM)	30,8	20,1
Starch (% DM)	6,8	19,9
Crude fat (% DM)	1,4	9,4
Ash (% DM)	7,45	4,52
Experimental diet chemical composition		
DM (%)	50,54	47,48
aNDFom (% DM)	34,33	33,85
Crude protein (% DM)	15,73	16,01
Non-fiber carbohydrate (% DM)	39,57	39,13
Ether extract (% DM)	4,13	4,77
GPM CT content (g CT/kg GM)	0	4,29

aNDFom, ash-corrected neutral detergent fiber; ADF, acid-detergent fiber; NFC, non-fiber carbohydrate; GPM, grape marc; CT, condensed tannin.

Supplementary Table S2. Dry matter intake, milk yield, and milk components of lactating Holstein dairy cows fed a diet supplemented with either grape marc (GPM) or a beet pulp: soy hulls mixture (control)

Parameter	Control	GPM	SE	*P*-value
DMI (kg/day)	25,83	26,61	0,93	0,57
Milk yield (kg/day)	39,87	36,22	1,22	0,07
Milk fat (%)	4,1	3,86	0,16	0,32
Milk protein (%)	3,34	3,43	0,04	0,13
Milk fat (kg/d)	1,62	1,38	0,85	0,1
Milk protein (kg/d)	1,32	1,23	0,05	0,19
SCC (Cells x1000)	83,15	76,12	24,99	0,85

Least square means reported for Control and GPM groups.

Supplementary Table S3. Nitrogen parameters of lactating Holstein dairy cows fed a diet supplemented with either grape marc (GPM) or a beet pulp: soy hulls mixture (control)

Parameter	Control	GPM	SE	*P*-value
Plasma urea nitrogen (mg/dL)	10,21	9,93	0,49	0,71
Milk urea nitrogen (mg/dL)	12,8	13,46	0,43	0,32
Urine urea (%)	0,06	0,02	0,027	0,34
Urine ammonia (%)	0,01	0,01	0,002	0,2
Urine crude protein (%)	5,36	6,06	0,68	0,49
Fecal nitrogen (%)	0,37	0,39	0,021	0,41
Fecal ammonia N (%)	0,03	0,03	0,002	0,29
Dietary nitrogen intake (g N/d)	619,4	606,3	32,75	0,78
Milk nitrogen output (g N/d)	193,4	194,3	16,28	0,97
Fecal nitrogen output (g N/d)	136,6	175	14,24	0,09
Urine nitrogen output (g N/d)	155,5	151,1	23,02	0,9
Retained nitrogen (g N/d)	133,9	86	46,23	0,48

Least square means reported for Control and GPM groups. Expressed as percent of DM.
SE, standard error.

Supplementary Table S4. SAS statistical outputs for the 127 Low-abundance enriched proteins identified in milk samples at significantly different relative-abundances collected from lactating Holstein dairy cows fed a diet supplemented with either grape marc (GPM; treatment) or beet pulp: soy hulls mixture (control)

Accession	Control	Treatment	SE	*P*-value	Accession	Control	Treatment	SE	*P*-value
A0A0A0MP92	1,1956	0,9802	0,1437	0,2404	G8JKX6	1,6328	3,0463	0,5807	0,0726
A0A0N4STN1	1,0696	1,7871	0,3788	0,0577	M0QVZ6	1,1854	1,3522	0,2208	0,4745
A0JNP2	1,4208	1,0118	0,2195	0,2393	M0QW03	1,0595	1,533	0,1587	0,0247
A2I7M9	1,5331	0,8859	0,2902	0,0942	P00711	1,5652	0,8785	0,269	0,0876
A5PJE3	1,1311	1,132	0,1399	0,9953	P00735	1,115	0,9094	0,106	0,1446
A5PJV3	1,0164	1,0182	0,09468	0,9861	P01035	1,165	0,6724	0,1256	0,0298
A6H7J6	1,0874	1,1295	0,1033	0,704	P01888	1,3053	0,8698	0,2078	0,0852
A6QLZ7	1,1274	1,113	0,1327	0,9134	P02662	1,1367	1,3664	0,1013	0,2514
A6QNZ7	1,0808	1,3017	0,171	0,1893	P02663	1,0861	1,3224	0,08903	0,1282
A6QPK0	2,4045	1,2404	0,5076	0,1789	P02666	1,0244	1,1343	0,0445	0,1944
A6QR11	1,1692	1,0228	0,1446	0,3863	P02668	1,0554	1,1162	0,07649	0,5148
A7E307	1,0114	1,0869	0,04826	0,1282	P02754	1,4676	0,7663	0,2452	0,0634
B8Y9T0	1,0511	1,2628	0,1226	0,0705	P02769	1,3043	0,6557	0,1793	0,0319
C6KGD8	1,0309	0,9678	0,06957	0,3035	P08037-2	1,1011	1,0328	0,1162	0,5303
E1B8N4	1,081	0,9479	0,1162	0,1905	P10096	1,0004	1,0393	0,01632	0,0159
E1BBX5	1,184	1,1243	0,1449	0,7271	P10152	1,258	0,9594	0,1365	0,2162
E1BDQ8					P10790	1,1943	1,0692	0,1616	0,5049
E1BGX8	1,0954	0,9898	0,1036	0,4082	P11151	1,0738	1,1148	0,09856	0,6767
E1BHY6	1,0678	0,9468	0,086	0,2314	P12763	1,2257	0,8874	0,1711	0,1152
E1BKM6	1,123	1,3749	0,09959	0,1897	P15467	1,1279	0,8944	0,09344	0,1485
E1BMJ0	1,2247	0,7714	0,1402	0,0591	P15497	1,0793	1,2048	0,1288	0,3271
E1BNL7					P17697	1,0361	1,4203	0,07266	0,0143
E1BNP8	1,1227	1,1209	0,1093	0,9904	P18892	1,1653	2,1284	0,3192	0,0151
F1MAV0	1,1318	1,1581	0,1397	0,8756	P24627	1,4131	1,0349	0,2261	0,2269
F1MCF8	1,4168	0,9161	0,2124	0,1361	P26201	1,4429	2,7866	0,5175	0,0395
F1MCX8	1,0927	1,1183	0,1175	0,8096	P26779	1,3232	1,3735	0,1854	0,8418
F1MFW9	1,0936	1,5197	0,1925	0,0727	P31096	1,1379	1,4378	0,1747	0,1153
F1MGG8	1,0806	0,9445	0,09264	0,2093	P34955	1,2482	1,0056	0,1521	0,2697
F1MIT3	1,4087	13,9726	3,5194	0,0222	P50397	1,1462	1,106	0,1182	0,7964
F1MJH1	1,0638	0,9086	0,08678	0,1438	P58073	1,0351	0,9046	0,09635	0,1183
F1MJV3	1,0305	0,9736	0,09459	0,6277	P61585	1,0708	1,0877	0,09693	0,8744
F1MLJ0	1,018	0,9764	0,08831	0,683	P62935	1,1074	1,1391	0,1217	0,8012
F1MLW8	1,354	0,9642	0,1963	0,1908	P79345	1,4079	0,9522	0,2239	0,1414
F1MM32	1,0653	0,7669	0,08752	0,0185	P80025	1,3304	0,9546	0,2315	0,1406

F1MMD7	1,1481	0,9296	0,1096	0,2026	P80195	1,2279	1,3268	0,1979	0,6155
F1MMW8	1,0902	1,7639	0,2346	0,0329	P80311	1,122	1,0306	0,1214	0,498
F1MNN7	1,0662	1,1364	0,1193	0,4391	P80457	1,1956	1,0643	0,1313	0,4768
F1MNV5	1,1545	0,7526	0,1305	0,0363	P81187	1,0538	0,929	0,06816	0,19
F1MQB0	1,0542	1,3022	0,108	0,0691	P81265	1,445	0,8519	0,2531	0,0896
F1MR60	1,0324	1,1056	0,04259	0,3803	P82943	1,2779	1,2086	0,2168	0,7706
F1MRD0	1,0562	1,2692	0,08244	0,0879	Q03247	1,0124	1,4312	0,08251	0,0022
F1MRM9					Q0IIG8	1,0284	1,0793	0,06885	0,403
F1MSZ6	1,1757	0,9529	0,1387	0,2063	Q0IIH5	1,2442	1,0682	0,1858	0,3827
F1MUP9	1,0208	1,2637	0,08073	0,0444	Q0P569	1,1832	1,1312	0,1655	0,7515
F1MUT3	1,2015	1,0666	0,1338	0,4734	Q0V8M0	1,0152	1,1628	0,0328	0,0457
F1MW07	1,0229	0,956	0,09544	0,5492	Q0VC21	1,2736	1,2772	0,2099	0,9864
F1MX50	1,2819	1,1216	0,2571	0,4681	Q0VCK8	1,0277	1,1064	0,05962	0,26
F1N045	1,1104	0,9648	0,097	0,2838	Q28065	1,0941	1,139	0,1168	0,7159
F1N0Q5	1,0348	1,1819	0,07506	0,105	Q29443	1,6671	0,8807	0,3244	0,0972
F1N5L2	1,0762	1,3461	0,1112	0,0813	Q2KIT0	1,149	1,0861	0,1209	0,6945
F1N5M2	1,3064	1,1852	0,1903	0,6209	Q2KIU3	1,1735	1,0318	0,1322	0,4297
F1N6D4	1,4708	2,8885	0,5652	0,0421	Q32KV6	1,0417	1,1275	0,09054	0,2899
F1N726	1,2823	0,8572	0,2024	0,1011	Q32PA1	1,1168	1,3208	0,135	0,2076
F6R3I5	1,0342	1,1902	0,06431	0,1031	Q3MHN2	1,0585	0,7316	0,08579	0,0124
G3MXB5	1,2058	1,1232	0,1681	0,6993	Q3SX32	1,0775	1,0519	0,1094	0,8179
G3N0S9	1,3064	1,3274	0,2272	0,9374	Q3SZZ9	1,1129	1,0902	0,1291	0,8738
G3N0V2	1,1773	1,3483	0,2153	0,4599	Q3ZBD1	1,1122	1,3998	0,1424	0,1123
G3N2D8	1,1353	1,4592	0,1806	0,1151	Q3ZCJ2	1,0342	1,1487	0,04507	0,2259
G3X7A5	1,2157	1,0554	0,1435	0,4369	Q4GZT4	1,1068	1,6246	0,2103	0,0208
G5E513	1,167	0,8508	0,1318	0,0908	Q6QRN6	1,0186	1,1984	0,05032	0,0683
G5E5H7	1,4612	0,7715	0,2448	0,0652	Q8WML4	1,4866	2,625	0,4724	0,0586
G5E5T5	1,1528	0,893	0,1246	0,1375	Q95114	1,2892	1,1041	0,2432	0,3692
G5E5V1	1,21	1,0062	0,1414	0,3297	Q95122	1,3136	1,0426	0,1977	0,2518
G5E6E9	1,6415	1,5438	0,3412	0,8709	Q9MZ06	1,1617	1,104	0,1522	0,7025
G5E6S0	1,5711	3,0634	0,5738	0,0555	Q9XSG3	1,0602	1,2884	0,1212	0,0852

Supplementary Table S5. Gene ontology annotation of milk proteins identified to be affected by treatment when lactating Holstein dairy cows were fed a diet supplemented with either grape marc (GPM) or beet pulp: soy hulls mixture (control)

Accession number	Protein	Biological Process	Molecular Function
Q03247	Apolipoprotein E	Anion transport, Biosynthetic process, Catabolic process, Cell differentiation, Cell growth, Cellular component biogenesis, Cellular component morphogenesis, Cholesterol metabolic process, Homeostatic process, Negative regulation of apoptotic process, Nervous system development, Protein metabolic process, Response to stress, Single-multicellular organism process	Enzyme activator activity, Lipid binding, Lipid transporter activity, Oxioreductase activity, Receptor binding, Transferase activity (transferring acyl groups)
Q3MHN2	Complement component C9	Cell-cell adhesion, Immune system process, Signal transduction	
P17697	Clusterin		
P18892	Butyrophilin subfamily 1 member A1	Cellular defense response, Cellular process, Exocytosis, Intracellular transport protein, Proteolysis, System development	Ubiquitin-protein ligase activity
F1MM32	Sulfhydryl oxidase	Cellular process	Oxioreductase activity, Protein disulfide isomerase activity
Q4GZT4	ATP-binding cassette sub-family G member 2	Catabolic process, Cellular process, N compound metabolic process, Nucleobase-containing compound metabolic process, Phosphate-containing compound metabolic process, Response to stimulus	ATPase activity (coupled to transmembrane movement of substances), Lipid transporter activity, Pyrophosphatase activity, Transmembrane transporter activity
F1MIT3	von Willebrand factor A domain-containing protein 8		
M0QW03	TPA: prolactin-like protein	Cellular process	Growth factor activity, Hormone activity
P01035	Cystatin-C	Proteolysis	Cysteine-type endopeptidase inhibitor activity, Protein binding
P02769	Serum albumin	Transport	
F1MMW8	Serum amyloid A protein	Cellular component movement, Localization, Locomotion, Response to external stimulus	
F1MNV5	Kininogen-1	Blood coagulation, Cellular calcium ion homeostasis, Cellular process, Regulation of biological process, Response to stress, Single-multicellular organism process	Cysteine-type endopeptidase inhibitor activity

P26201	Platelet glycoprotein 4	Cell adhesion, Cellular process, Macrophage activation	Receptor activity
F1N6D4	Sodium-dependent phosphate transport protein 2B	Anion transport, Cellular process, Homeostatic process, Phosphate ion transport	Cation transmembrane transporter activity
F1MUP9	synaptic vesicle membrane protein VAT-1 homolog	Apoptotic process, Carbohydrate metabolism process	Oxioreductase activity
Q0V8M0	Protein KRI1 homolog		

Accession number	Protein	Cellular Process	Protein Class
Q03247	Apolipoprotein E	Extracellular space, Macromolecular complex	
Q3MHN2	Complement component C9		
P17697	Clusterin		
P18892	Butyrophilin subfamily 1 member A1		Ubiquitin protein ligase
F1MM32	Sulfhydryl oxidase	Golgi apparatus, Cytoplasm, Extracellular space, Integral to membrane	Oxidase
Q4GZT4	ATP-binding cassette sub-family G member 2	Apical part of cell, Integral to membrane, Plasma membrane, Protein complex	ATP-binding cassette (ABC) transporter
F1MIT3	von Willebrand factor A domain-containing protein 8		
M0QW03	TPA: prolactin-like protein		Growth factor, Peptide hormone
P01035	Cystatin-C		Cysteine protease inhibitor
P02769	Serum albumin		Transfer/ carrier protein
F1MMW8	Serum amyloid A protein	Extracellular space	Apolipoprotein, Defense/immunity protein, Transporter
F1MNV5	Kininogen-1	Extracellular space	

P26201	Platelet glycoprotein 4		Receptor
F1N6D4	Sodium-dependent phosphate transport protein 2B	Apical part of cell, Cell projection, Cytoplasm, Organelle, Plasma membrane	Enzyme modulator, Transporter
F1MUP9	synaptic vesicle membrane protein VAT-1 homolog		Dehydrogenase, Reductase
Q0V8M0	Protein KRI1 homolog		
